# A Microwell Platform for Characterizing the Dynamic Response of Corneal Keratocytes to Biochemical and Biophysical Cues

**DOI:** 10.3390/mi17070783

**Published:** 2026-06-27

**Authors:** Tarik Z. Shihabeddin, Nathaniel S. Tjahjono, Divya Subramanian, Abbas Rizvi, Miguel Miron-Mendoza, Victor D. Varner, David W. Schmidtke

**Affiliations:** 1 Department of Bioengineering, University of Texas at Dallas, Richardson, TX 75080, USA; tarik.shihabeddin@utdallas.edu (T.Z.S.); nathaniel.tjahjono@utdallas.edu (N.S.T.); divya.subramanian@utdallas.edu (D.S.); abbasanengineer@gmail.com (A.R.); vdv@utdallas.edu (V.D.V.); 2 Department of Ophthalmology, University of Texas Southwestern Medical Center, Dallas, TX 75390, USA; miguel.miron@utsouthwestern.edu; 3 Department of Biomedical Engineering, University of Texas Southwestern Medical Center, Dallas, TX 75390, USA

**Keywords:** corneal keratocytes, fibronectin, collagen fibrils, time-lapse imaging, topography

## Abstract

The interaction of corneal keratocytes with biochemical (e.g., composition, growth factors) and biophysical (e.g., topography) cues present in the cornea regulates their morphology during normal homeostasis and wound healing. In this study, we developed a novel method of fabricating substrates with micropatterns of Type I aligned collagen fibrils in a 6-well format that allowed for time-lapse imaging of dynamic changes in keratocyte morphology. Culturing keratocytes on aligned collagen fibrils in the presence of platelet-derived growth factor BB (PDGF-BB) allowed us to characterize the dynamics of cell alignment and migration. To investigate the roles of topography and protein composition on the dynamic features of cell spreading, cell protrusions, and cell motility, we cultured keratocytes on either hydrophobic-coated glass, aligned collagen fibrils, or monomeric collagen with or without a fibronectin coating. The presence of a fibronectin coating delayed the formation of cell protrusions during spreading on all of the substrates tested (e.g., Aquasil-coated glass, monomeric collagen, aligned collagen fibrils), while the presence of aligned collagen fibrils resulted in a ~2-fold reduction in the cell spreading area. The experimental platform developed here allows for parallel experiments and real-time imaging and thus providing a valuable new tool to study the dynamic activity and cell–substrate interactions of corneal keratocytes. This approach will allow for systematic screening of the response of keratocytes and other cell types (e.g., tenocytes, cardiomyocytes, cancer cells) that normally are exposed to aligned collagen topographies.

## 1. Introduction

Corneal blindness is the fourth leading cause of blindness worldwide [1]. Both disease and injury can cause improper healing of the cornea and the deposition of scar tissue that significantly impairs patient vision and potentially leads to blindness [2]. In the normal cornea, quiescent cells called keratocytes reside between lamellae (layers of aligned collagen fibrils in the corneal stroma) and have a stellate morphology [3]. Following corneal injury, multiple growth factors such as transforming growth factor beta (TGF-β), platelet-derived growth factor BB (PDGF-BB), fibroblast growth factor (FGF), and insulin-like growth factor 1 (IGF-1) are secreted into the wounded area [4,5,6,7]. Similarly, fibronectin is also secreted into the wound area [8,9], and not only forms a temporary scaffold in the early stages of injury but is also organized into tracks that are used by other trailing cells to form interconnected lines of cells [5,10]. Exposure to these cues transform the quiescent keratocytes into more activated repair phenotypes, such as fibroblasts and/or myofibroblasts [7,11], and change their stellate morphology into a distinct set of cell morphologies (e.g., PDGF-BB induces an elongated morphology and TGF-β induces a polygonal morphology) [12,13]. However, these growth factors do not operate in isolation, and changes in cell morphology are strongly influenced by many properties of the extracellular matrix (ECM), such as the ECM composition, topography and stiffness.

The composition (biochemical cue) and topography (structural cue) of the corneal stroma ECM influences cell morphology and behavior primarily through integrin-mediated adhesion and mechanotransduction signaling. For example, the composition of the ECM can also modulate keratocyte behavior given that specific ECM proteins promote distinct integrin binding interactions (e.g., α_5_β_1_—fibronectin binding, α_2_β_1_—collagen binding) and modulate the size, number, and organization of focal adhesions (FAs) which in turn influence cell morphology, spreading, and motility [14,15]. Similarly, topographic features can modulate corneal keratocyte spreading [16], focal adhesion (FA) formation [16,17,18], alpha smooth muscle actin (α-SMA) expression [19,20], and TGF-β-induced myofibroblast differentiation [13,19].

One challenge in understanding how topography influences the dynamic behavior of corneal keratocytes is recapitulating the highly organized aligned collagen fibril architecture. To address the challenge of fabricating aligned collagen fibrils that mimic the architecture of the cornea and other tissues (e.g., tendons), a number of strategies have been developed. Some of the more popular fabrication methods include electrospinning [21], electrochemical fabrication [22], freeze drying [23], magnetic fields [24], molecular crowding [25], microfluidic shear flow deposition [26,27,28,29], flow-induced crystallization [30,31], and wet spinning [30,31]. The advantages and disadvantages of these methods have been reviewed previously. Our group has focused on the use of the microfluidic shear flow deposition method due to its simplicity and ability to fabricate micropatterns of aligned collagen fibrils with different geometries on transparent and elastic substrates [32].

Previous studies have shown that cellular morphology and migration are intimately linked to their biological function. For example, cell shape has been shown to not only regulate normal biological processes such as proliferation [33], differentiation [34], apoptosis [33], gene expression [35], and tissue morphogenesis during development [36], but also to be important in several pathologies. For example, changes in cell morphology often precede cancer cell malignancy [37], while changes in the morphology and stiffness of red blood cells may provide specific signatures of the neurodegenerative pathologies [38]. In addition, recent studies have shown how (i) topography-induced shapes correlate with shape-induced cell signaling to different phenotypes in bone marrow-derived mesenchymal stem cells [39], and (ii) that combining single-cell morphological profiling with image-based learning can be used to predict macrophage phenotype and intracellular IL-10 levels [40]. Likewise, cell migration is a fundamental process in a number of physiological and pathological processes such as angiogenesis [41], embryonic development [42], cancer metastasis [43], and wound healing [44].

Although many in vivo and in vitro studies have improved our understanding of how various biochemical and biophysical cues affect keratocyte morphology and behavior during wound healing, there are still significant gaps in our knowledge. One challenge is that most studies examining keratocyte behavior have been based on fixing, fluorescently staining, and imaging cells at distinct time points. Although fixed-cell assays are relatively simple to perform, a limitation is the loss of information regarding cellular dynamics in response to transient or long-term exposure to growth factors and topography. Furthermore, imaging artifacts may occur due to cell fixation and permeabilization. Thus, there is an increasing desire for live-cell imaging in order to characterize cell shape dynamics and polarization of corneal keratocytes in response to simultaneous exposure to both biophysical (e.g., topography of aligned collagen fibrils) and biochemical cues (e.g., fibronectin).

We previously showed that primary rabbit corneal keratocytes cultured on fibronectin-coated surfaces become activated by 48 h, as measured by α-SMA expression, the presence of stress fibers, and a spread morphology, but then transition into a more quiescent, stellate phenotype lacking these features by 120 h [20]. The activation of the keratocytes was lower when aligned collagen fibrils were present. A limitation of our previous system was that the fabrication of the aligned collagen fibrils substrates required large rectangular glass coverslips which limited us to (i) static imaging experiments that overlooked the dynamic changes during keratocyte activation, or (ii) time-lapse imaging of a single condition. As such, it was difficult to determine if the same cells that were activated on fibronectin returned to more quiescent morphology, or if they went through apoptosis and the cells that were stellate by 120 h were simply cells that had not been activated. In addition, with our previous system, we could not perform time-lapse analysis of cell orientation on the aligned collagen fibrils over time. Thus, the goal of this study was to develop a method of fabricating aligned collagen fibrils on substrates compatible with time-lapse imaging in a 6-well format which will allow us to characterize the dynamic changes in keratocyte behavior in response to simultaneous exposure of both biophysical and biochemical cues.

## 2. Materials and Methods

### 2.1. Fabrication of PDMS Microfluidic Devices for Aligned Collagen Fibril Deposition

Photoresist templates were fabricated utilizing a protocol similar to that previously published [20,45]. Initially, photoresist templates were fabricated by spin coating KMPR 1050 photoresist (Microchem; Westborough, MA, USA) onto a four-inch silicon wafer. The photoresist–silicon wafer was placed onto a digital aluminum hot plate (Torrey Pines Scientific; Carlsbad, CA, USA) at 100 °C for 15 min to soft bake and then allowed to cool for 1 min. The wafer was exposed to UV light through a chrome mask with a mask aligner (Karl Suss; Munich, Germany) to obtain the desired pattern. Following UV exposure, the wafer was hard baked for 3 min at 100 °C and cooled for 1 min. The wafer was then placed in SU-8 developer for 3 min with constant shaking, dried with nitrogen gas, and a profilometer (Veeco Instruments; Oyster Bay, NY, USA) was used to ensure the channel height was 50 µm. The photoresist patterns were then coated with a thin layer of Tridecafluoro-1,1,2,2-tetrahydrooctyl)methyldichlorosilane (Gelest Inc.; Morrisville, PA, USA), to facilitate detachment of the polydimethylsiloxane (PDMS) molds without removing the photoresist patterns, by exposing the patterns to the fluorosilane vapor for 4 h.

The fabrication of PDMS microfluidic devices followed an established procedure [20,45]. Briefly, 30 g of Sylgard 184 Silicone Elastomer (Dow Corning; Midland, MI, USA) was combined with 3 g of the curing agent, mixed thoroughly, and carefully poured onto the wafer until the photoresist pattern was completely covered. The wafer was then placed in a vacuum chamber for 1 h at room temperature to remove any air bubbles from the uncured PDMS, and plastic elbows (Nordson Medical; Loveland, CO, USA) were placed on the channel inlet ports before being cured in an oven for 40 min at 80 °C. The cured PDMS stamps with the desired microfluidic channel design were then cut out with an X-Acto knife and trimmed so that they could fit into a 6-well plate. Outlet ports were created by using a 3 mm biopsy punch, PDMS plugs were removed from the plastic elbows, and Scotch Magic Tape was used to remove particulates from the PDMS stamps.

### 2.2. Glass Slide Preparation

Circular glass coverslips (#1.5, 30 mm diameter, Fisher Scientific; Hampton, NH, USA) were carefully loaded into staining racks (Electron Microscopy Sciences; Hatfield, PA, USA) and initially rinsed three times with ultrapure water before being submerged in a 28% Nitric Acid solution for 1 h. Afterwards, coverslips were rinsed at least three times with ultrapure water before being placed in a vacuum oven at 80 °C until fully dried. Once dry, the coverslips were allowed to cool to room temperature. The glass coverslips were made hydrophobic by submerging them in an aqueous 1% Aquasil solution (Fisher Sci; Hampton, NH, USA) for 15 s. Following Aquasil treatment the coverslips were immediately submerged in ultrapure water and washed three times with ultrapure water before drying in a vacuum oven at 80 °C. After drying, the Aquasil-coated glass coverslips were cooled to room temperature and stored for subsequent integration into 6-well plates.

### 2.3. Fabrication of Customized 6-Well Plates

**Figure 1:**
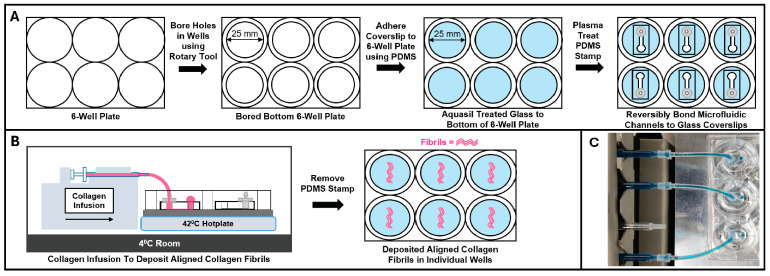
Method for depositing aligned collagen fibrils into the wells of a 6-well plate. The fabrication of the aligned collagen fibril substrates in the 6-well plate format was a multistep process. In the first step of the process (**A**) a 6-well plate was modified by boring a hole in each well, and next an Aquasil-coated glass coverslip was bonded to the bottom of each well, and a plasma-treated PDMS microfluidic chip was reversibly bonded to the glass coverslip. (**B**) In the second step of the process the modified 6-well plate was placed on a hot plate, syringes loaded with unpolymerized collagen solutions were connected to three of the microfluidic chips, and the chilled collagen solutions were perfused through the microchannels at well-defined flow rates. As the collagen solution polymerizes, aligned collagen fibrils are formed and deposited on the hydrophobic glass surface. (**C**) Image of the syringes—microfluidic chip connections for three wells. The syringes, connecting tubing and microfluidics flow channels, were filled with blue food coloring for illustration.

We fabricated customized 6-well plates containing the Aquasil-coated glass coverslips to promote aligned collagen fibril deposition and subsequent cell culture (Figure 1). Initially a rotary tool was used to bore out a 25 mm opening in the center of each well of a 6-well plate (Figure 1A). The 6-well plate was rinsed clean, dried, and then placed face-down so that the bottom of the plate was facing upward. Circular Aquasil-treated glass coverslips were then placed carefully along the remaining lip of each well in order to cover the bored holes. PDMS was then pipetted along the lip at the glass–plastic interface, and the 6-well plate was placed in an 80 °C oven and cured for 40 min.

### 2.4. Preparation of Protein-Coated Substrates

We functionalized the Aquasil-coated coverslips in the customized 6-well plates by modifying a well-established procedure for fabricating aligned Type I collagen fibrils [20,45]. Initially cleaned PDMS stamps were placed on their sides and exposed to an air plasma (Harrick PlasmFlo chamber; Ithaca, NY, USA) for one minute at a high radio frequency (RF). Within one minute of plasma exposure, a PDMS stamp was placed into each well of the Aquasil-coated glass-bottom 6-well plate. The 6-well plate was then placed on a hot plate at 40 °C for 30 min (Figure 1B). Meanwhile, a 3 mL collagen solution (1.6 mg/mL) was prepared by mixing the following components: 200 µL of 10× MEM, 200 µL of 0.1 M NaOH, 1000 µL of 1× MEM, and 1600 µL of stock 3.0 mg/mL Type I PureCol Bovine Collagen. The pH of the 3 mL solution was adjusted to 7.35 or 7.5 by adding small aliquots of 0.1 M NaOH as needed. The collagen solution was cooled to 4 °C in a cold room before loading it into 1 mL syringes (BD Bioscience; Franklin Lakes, NJ, USA). After connecting the collagen loaded syringes to 3 of the microfluidic chambers in the 6-well plate (Figure 1C) the syringes were loaded into a 10 channel syringe pump (KD Scientific; Holliston, MA, USA) and the collagen solution was perfused at a shear rate of 150 s^−1^ (5.1 µL/min) or 225 s^−1^ (7.65 µL/min) for 30 min. After infusion, stamps were removed from the 6-well plate and ultrapure water was used to briefly rinse the collagen lines and remove excess collagen solution. The plates were placed on a hot plate at 40 °C to dry and then sterilized by UV exposure for 15 min in the biosafety cabinet. To deposit uniform coatings of monomeric collagen, a 1 mL solution of unpolymerized collagen (50 μg/mL) was added to the Aquasil-coated coverslips and allowed to adsorb to the substrate for 2 h at room temperature. The substrates were then gently washed with 1× phosphate-buffered saline (PBS) before being dried.

To investigate the effects of fibronectin on keratocyte behavior, solutions of fibronectin (50 μg/mL) were pipetted into the 6-well plates and allowed to adsorb for 2 h at room temperature onto uncoated glass, monomeric collagen, or aligned collagen fibrils. The fibronectin solutions were prepared by diluting a stock solution of fibronectin (EMD Millipore) with PBS to the desired concentration. Following the 2 h incubation, the fibronectin solution was aspirated from the substrates, and the substrates were washed with PBS before cell seeding.

### 2.5. Primary Keratocyte Cell Isolation and Cell Culture

Primary corneal keratocytes (also known as normal rabbit keratocytes or NRKs) were isolated from the eyes of New Zealand White Rabbits (Pelfreez Arkansas; Rodgers, AR, USA) and cultured as described [20,45,46] To maintain a quiescent phenotype, the NRKs were cultured for 4 days at 37 °C in T25 flasks and serum-free media that consisted of Dulbecco’s modified Eagle’s medium (DMEM) supplemented with 100 μM nonessential amino acids (Invitrogen; Carlsbad, CA, USA), 100 μg/mL ascorbic acid, 1% RPMI vitamin mix, and 1% PenStrep (Invitrogen; Carlsbad, CA, USA). For all experiments, first passage NRKs were cultured in serum-free media for 4 days prior to seeding onto the different substrates.

At the beginning of an adhesion experiment, NRKs were: detached from the culture flasks by adding 2 mL of a 0.25% trypsin/EDTA solution to the flask, placed in a 37 °C incubator for 3 min, agitated gently, treated with 8 mL of trypsin inhibitor (Sigma-Aldrich; St. Louis, MO, USA), centrifuged at 1500 rpm for 4 min, and resuspended to a concentration of 20,000 cells/mL in serum-free media. Next, NRKs were seeded onto the different substrates by adding 2 mL of the keratocyte suspension to each well. For some experiments, platelet-derived growth factor BB (PDGF-BB) (Gibco; Waltham, MA, USA) was added to the media to obtain a final concentration of 50 ng/mL [20,45].

### 2.6. Time-Lapse Video Microscopy

Cells were cultured at 37 °C under 5% CO_2_ using a Zeiss stage incubator system during time-lapse experiments. For measurement of cell orientation, migration, and morphology, microscopic images were captured every hour for 5 days using a Zeiss 10× EC-Plan NEOFLUAR objective (NA = 0.3) on a Zeiss AxioObserver Z1 Phase Contrast Microscope equipped with an Orca Flash 4.0 monochrome CMOS camera. For fluorescent imaging of nuclei and actin, a Zeiss AxioObserver Z1 Inverted Microscope using a 20X Plan-Apochromat (NA = 0.8) and/or a 63X Plan-NeoFluor (NA = 1.4) objective was used.

### 2.7. Immunohistochemistry

Cells were fixed using a 3% paraformaldehyde solution in PBS for 15 min at room temperature and washed three times for 20 min each with PBS. To label cells for nuclei and F-actin, cells were permeabilized by treatment with 0.5% triton X-100 for 15 min, washed three times for 10 min each with PBS, blocked for 1 h at room temperature with 1% bovine serum albumin (BSA, fraction V) in PBS, and washed again with PBS for 20 min three additional times. Cell actin filaments and nuclei were labeled by incubation with Alexa Fluor 647 Phalloidin (1:200 dilution, Molecular Probes, Eugene, OR, USA) and with 4′,6-diamidino-2-phenylindole (DAPI) solution (1:1000 dilution) for 1 h followed by three 10 min washes with PBS. Each condition was evaluated in at least 3 independent experiments.

### 2.8. Alignment, Migration, and Solidity Analysis

The degree of fibril and keratocyte alignment was measured using the ImageJ Directionality plugin (Version 2.0) to perform a Fourier component analysis followed by the calculation of an orientation index (OI) as described previously [20,45]. An OI value of 100% indicates keratocyte co-alignment in the direction of the aligned collagen fibrils while 0% represents random keratocyte alignment. Cell migration analysis was performed using Image-Pro Premier image analysis software (Media Cybernetics; Rockville, MD, USA). Time-lapse CZI files from the Zeiss Zen software were opened directly in Image-Pro Premier, cells were manually tracked throughout the entire time-lapse video, and data files containing cell tracking information (i.e., cell distances, speeds, etc.) were exported and compiled for further analysis. Solidity analysis was done using the Zeiss Zen software. Time-lapse CZI files were opened, and manual measurement of cell area and cell convex hull area was performed. The obtained values were compiled, and solidity values were calculated for further analysis.

### 2.9. Statistical Analysis

Statistical analysis of the data was performed with GraphPad Prism 11.0.2 (GraphPad; San Diego, CA, USA). Data represents mean ± standard deviation from at least three experimental replicates. When appropriate either a *t*-test, a one-way, or a two-way ANOVA with a Tukey post hoc test was used to determine statistical differences, and *p*-values are reported in the figure captions.

## 3. Results and Discussion

### 3.1. Fabrication of the 6-Well Platform for Depositing Aligned Collagen Fibrils

The extracellular matrix (ECM) is known to provide a number of biophysical (e.g., topography, elasticity, dimensionality) and biochemical (e.g., ECM proteins, growth factors) cues that modulate cell behavior. The integration of these competing signals by cells leads to a complex, dynamic response that changes over time. Development of assays to untangle the roles of the competing signals requires high throughput culture systems that closely mimic the native microenvironment and provide real-time imaging of their dynamic responses. Therefore, we designed a novel technique that allows for simultaneous live-cell imaging of how cells respond to substrates that incorporate several different stimuli (i.e., topography, proteins, growth factors, etc.). As proof of concept, our first goal was to develop substrates that mimic the native topography of the cornea by depositing aligned collagen fibrils into individual wells of a 6-well plate. This was accomplished by (i) permanently bonding Aquasil-coated circular glass coverslips to the bottom of modified 6-well plates; (ii) reversibly bonding our novel microfluidic devices to the glass coverslips; (iii) placing the modified 6-well on a hot plate; and (iv) perfusing unpolymerized collagen solutions through the microfluidics devices in order to deposit aligned collagen fibrils onto the hydrophobic glass coverslips (Figure 1). Figure 1B shows the dimensions of the microfluidic channels, while Figure 1C shows side and top view images of the actual devices.

### 3.2. Optimization and Characterization of the Deposited Aligned Collagen Fibrils

**Figure 2:**
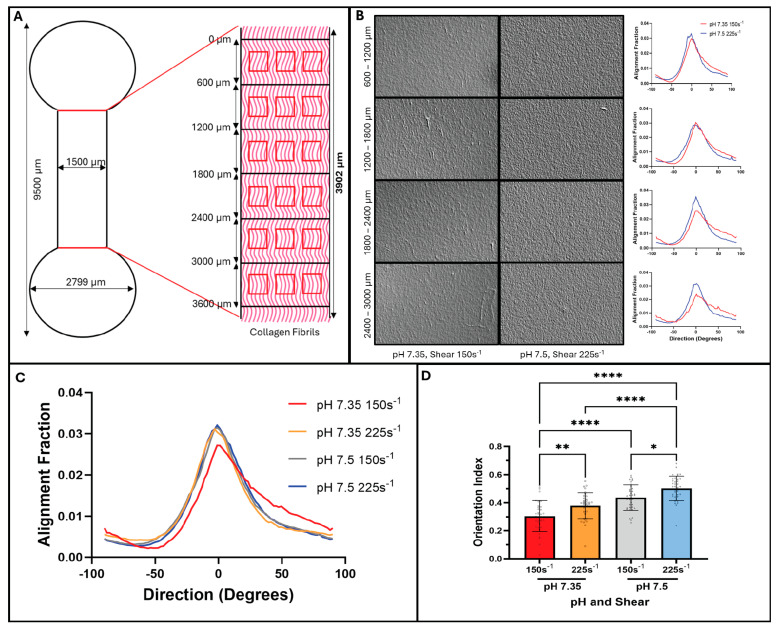
Effects of shear rate and pH on collagen fibril alignment. (**A**) Schematic of how the alignment of the collagen fibrils were analyzed over the entire channel length. (**B**) Representative DIC images of aligned collagen fibrils at two different fabrication conditions and the corresponding directionality plots. Fibrils are oriented vertically. (**C**) Plots of the average directionality in the main part of the channel at the different pH and shear rate conditions (*n* = 4 substrates). (**D**) Plot of the orientation index at two different pH and shear rates. Data represents mean ± standard deviation for four experimental repeats. Each data point can be visualized as a single red square in section A from 600 μm to 3000 μm along the entire channel. Data were analyzed using a two-way ANOVA followed by a Tukey post hoc test. A significance level of *p* < 0.05 was used for all the tests. ns *p* > 0.05; * *p* < 0.05; ** *p* < 0.01; **** *p* < 0.0001.

Previously we reported that the density and degree of collagen fibril alignment depend on the shear rate at which the chilled Type I collagen solution is perfused [45]. To ensure reproducible fabrication of aligned collagen fibrils in our new microfluidic device, we varied both the shear rate and the pH of the collagen solution. To determine whether the alignment of collagen fibrils was dependent on its position in the microfluidic channel, we divided the substrate area containing the deposited fibrils into six sections that were 600 μm long by 1500 μm wide (Figure 2A) and acquired differential interference contrast (DIC) images of the collagen fibrils in the different sections (Figure 2B). From the DIC images we then measured the degree of alignment and the orientation index (OI) of the collagen fibrils within each section. As shown in Supplementary Figure S1, the alignment for the sections closest to the inlet (0–600 μm) and outlet ports (3000–3600 μm) were lower than the main part of the channel (600–3000 μm). Thus, when examining the effects of shear and pH on the degree of fibril alignment (Figure 2C), we calculated an average OI for the main part of the channel. In general, a pH of 7.5 resulted in a higher OI compared to pH of 7.35 for both shear rates (Figure 2D), and a shear rate of 225 s^−1^ resulted in a higher OI than 150 s^−1^ at both pH conditions. Thus, all subsequent experiments involving aligned collagen fibrils were performed at a pH of 7.5 and a shear rate of 225 s^−1^. These OI values and directionality values for the deposited collagen fibrils are in the same range as those we have previously reported, which were fabricated with larger microfluidic devices [20,45,46].

### 3.3. Dynamics of Keratocyte Behavior on Aligned Collagen Fibrils in the Presence of PDGF-BB

**Figure 3:**
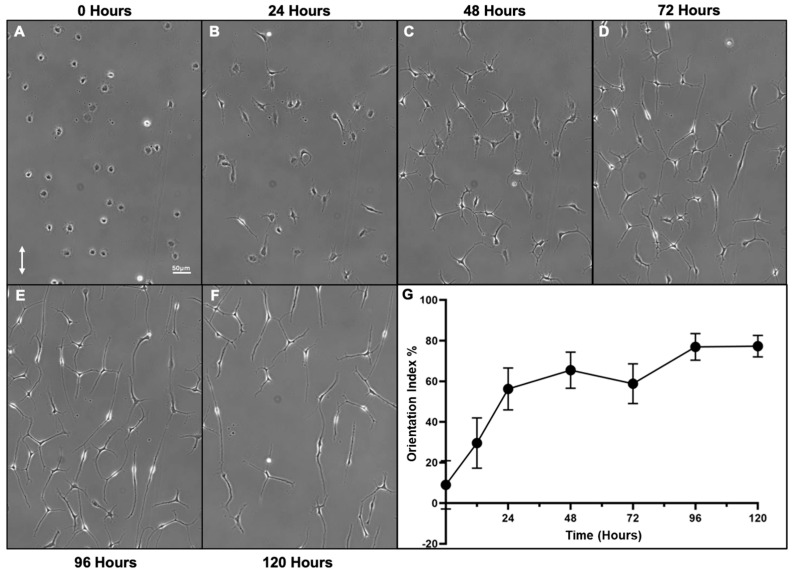
Dynamics of keratocyte morphology and alignment on aligned collagen fibrils in the presence of PDGF-BB. Primary rabbit keratocytes were cultured on aligned collagen fibrils for 120 h in serum-free media containing 50 ng/mL PDGF-BB and phase contrast time-lapse microscopy was performed with a 10× objective with images acquired every hour. (**A**–**F**) Six phase contrast images from a representative time-lapse sequence are shown (see Video S1 in Supplementary Data). The white arrow shows the direction of the collagen fibrils. (**G**) Plot of the average orientation index of the keratocytes relative to the fibrils as a function of time. (*n* = 3 experimental replicates, 30 cells per data point).

**Figure 4:**
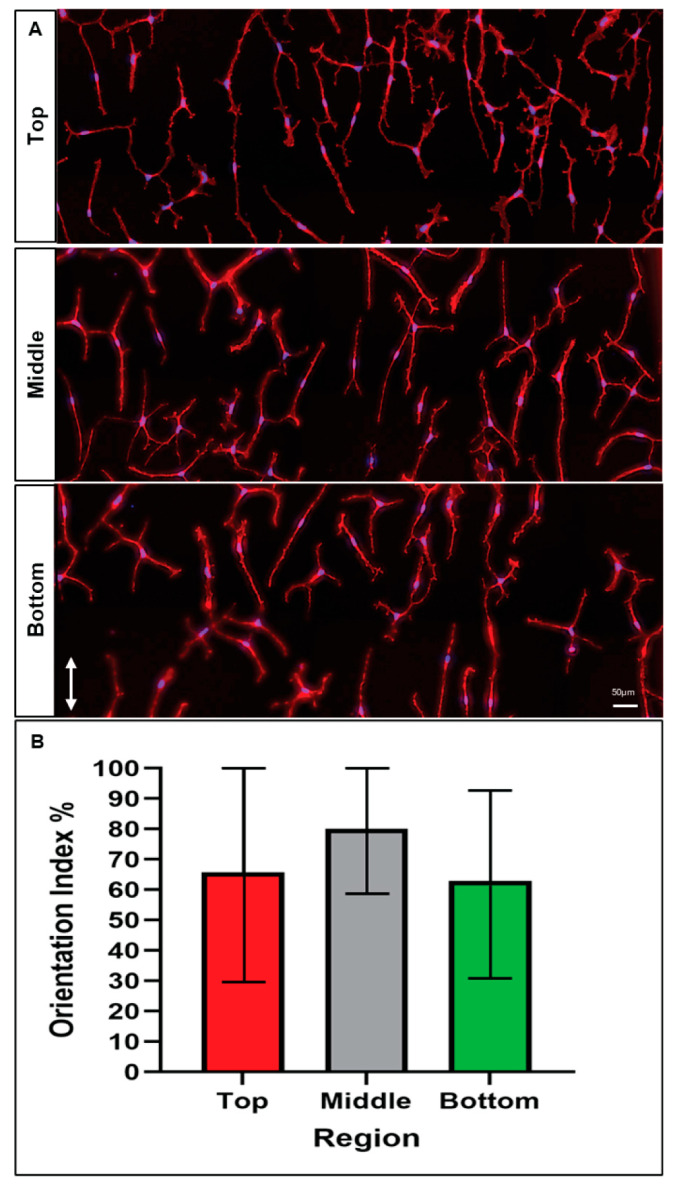
Cell alignment at different micropattern locations. (**A**) Representative fluorescent images of primary keratocytes cultured for 120 h in the presence of PDGF-BB on micropatterns of aligned collagen fibrils at the top, middle, and bottom regions of the micropatterns. The white arrow shows the direction of the collagen fibrils. (**B**) Quantitative analysis of the alignment of the above keratocyte images. Cell orientation index was measured at three different positions in each region. (*n* = 3 experimental replicates, 60 cells were analyzed for each condition). A one-way ANOVA followed by a Tukey post hoc test was performed. A significance level of *p* < 0.05 was used for all the tests; however, no significant differences were found. Error bars are S.D.

Previously we reported that when primary keratocytes (NRKs) are cultured on aligned collagen fibrils for 48 h in serum-free media supplemented with PDGF-BB, they align in the direction of the fibrils [45]. To validate that keratocytes will behave in a similar way when cultured on the aligned collagen fibrils deposited in the 6-well plates, we cultured NRKs under similar conditions and set up a time lapse for 120 h (Figure 3, Supplementary Video S1). As shown in Figure 3A, when cells initially attached to the surface, they had a rounded or discoid morphology, with little to no alignment in the direction of the aligned collagen fibrils. However, within 12–24 h the cells became elongated with a polarized morphology that aligned in the direction of the aligned collagen fibrils (Figure 3B) and began to form dendritic cell processes with neighboring cells. At 48 h, cell extensions continued to elongate, and more alignment occurred. By 72 h, all the cells had elongated extensions, and many seemed to align in the direction of the fibrils. At 96 h, the cells had either a branched or bipolar morphology with most of the cells’ major axes aligned in the direction of the collagen fibrils. A plot of the orientation index (OI) vs. time (Figure 3G) shows that most of the cell alignment occurs during the first 48 h. The degree of cell alignment observed at the 48 h mark is similar to what we have observed previously for NRKs cultured in the presence of PDGF-BB on aligned collagen fibrils [45]. At the final time point (120 h), the cells were fixed, stained, and imaged throughout the aligned collagen fibril micropattern to determine whether the cell alignment was dependent on its location on the micropattern. As shown in Figure 4B, the average OI values for cells in the top and bottom locations of the micropattern was ~0.6, while the OI in the middle location was ~0.8. These fluorescent imaging results suggest that the overall alignment of cells was fairly consistent throughout the micropattern. Taken together the results of the dynamic and static imaging suggest that our process for depositing aligned collagen fibrils is equivalent to our previous method and also provides new data on the temporal dynamics of corneal keratocyte alignment on aligned collagen fibrils.

**Figure 5:**
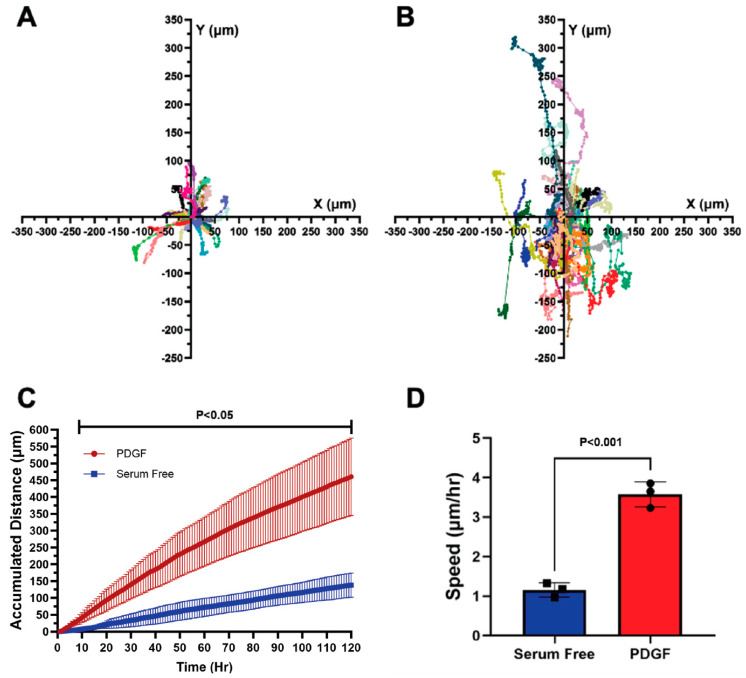
Effect of PDGF-BB on keratocyte motility on aligned collagen fibrils. Cell tracking plot displaying the individual cell paths from their origin as a function of distance in the x-y directions for 30 cells on aligned collagen fibrils in (**A**) serum-free media and (**B**) media containing 50 ng/mL PDGF-BB from three experimental replicates. Each colored line represents the actual spatial coordinates of one cell track. (**C**) Quantification of the accumulated displacement over time. Each data point represents the mean ± S.D. of the accumulated distance traveled from 30 cells over 120 h from *n* = 3 experimental replicates. (**D**) Plot of the cell motility speed. Each data point represents the mean ± S.D. of the cell speed from N= 3 replicates. Accumulated distance data were analyzed using a two-way ANOVA with a significance level of *p* < 0.05 followed by a Tukey post hoc test. Speed data were analyzed using a *t*-test with a significance level of *p* < 0.05.

We also analyzed the dynamic motility of the NRKs on the aligned collagen fibrils in the presence and absence of PDGF-BB (Supplementary Videos S1 and S2). Figure 5 shows representative cell tracking plots for 30 cells over 120 h on aligned collagen fibrils in serum-free media and media containing PDGF-BB. Visually, one can see that in serum-free media NRKs migrated randomly and typically traveled only a small distance from their starting point with a maximum distance of ~100 μm (Figure 5A). In contrast, when PDGF-BB was present, keratocytes not only moved further away from their starting point (~350 μm) but also generally in the direction of the aligned collagen fibrils (Figure 5B). The differences in the cell trajectory between the PDGF-BB and serum-free treated cells suggest that the topography of the aligned collagen fibrils has a bigger role in influencing the directionality of the cells when cells are more motile. Using the cell trajectories, we also computed the accumulated travel distance (Figure 5C) and the cell speed (Figure 5D). Quantification of average cell speed showed that PDGF-BB-treated cells migrated ~3.5 times more quickly than their counterparts in serum-free conditions. The increases in distance traveled and cell motility in the presence of PDGF-BB as compared to basal media are consistent with previous reports that examined keratocyte motility on elastic substrates [47] or in compressed collagen matrices [12].

### 3.4. Effects of Topography and Fibronectin on the Dynamics of Keratocyte Morphology

Earlier studies by our groups observed that exposure of corneal keratocytes to adsorbed fibronectin transiently induced them to become activated as indicated by a change in their morphology, the presence of stress fibers, and α-SMA expression levels [20]. The extent of keratocyte activation by fibronectin was affected by both the length of cell culture (2 vs. 5 days) and the topography of the substrate (i.e., fibronectin-coated aligned collagen fibrils vs. fibronectin-coated glass). A limitation of this previous study was that cells had to be fixed and stained at distinct time points to assess cell morphology and activation.

**Figure 6:**
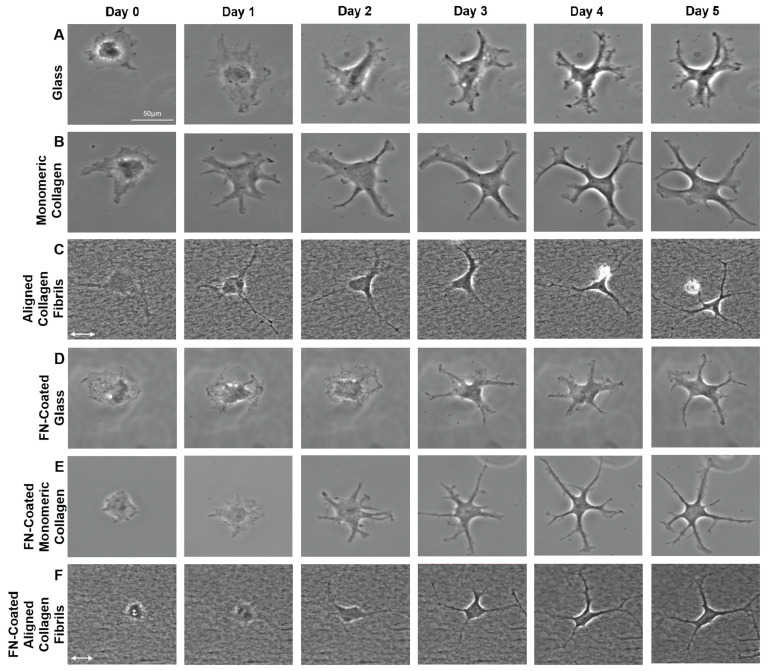
Effects of topogography and compostion on keratocyte morphology dynamics. Primary rabbit keratocytes (NRKs) were cultured in serum-free media on three different substrates (uncoated glass, monomeric collagen, or aligned collagen fibrils) (**A**–**C**) without and (**D**–**F**) with a fibronectin coating. The white arrow shows the direction of the collagen fibrils. Phase contrast time-lapse microscopy was performed with a 10× objective with images collected every hour. For each substrate six phase contrast images from a representative time-lapse sequence are shown (see Videos S2–S7 in Supplementary Materials).

To better understand the dynamics of the response of keratocytes to topography in the absence and presence of adsorbed fibronectin, we employed our 6-well plate assay and conducted a set of time-lapse imaging experiments. Figure 6 shows representative phase contrast images extracted from time-lapse videos of primary keratocytes cultured on six different substrates in serum-free media. When keratocytes were cultured on glass (Figure 6A), monomeric collagen (Figure 6B), or aligned collagen fibrils (Figure 6C) without any fibronectin, keratocytes transformed from a spherical morphology to a spread morphology during the first 24 h (see Supplementary Videos S2–S4). Keratocytes adhered to the glass or monomeric collagen had a similar size, while keratocytes on the aligned collagen fibrils had a significantly smaller spreading area. After 24 h, differences were observed in the keratocyte morphology on the three different substrates. Keratocytes on glass had a spread morphology and a large area, while keratocytes on monomeric collagen had a large area with thick branches extruding from the cell body. In contrast keratocytes on the aligned collagen fibrils had a smaller cell area with thinner and longer branches coming from the cell body. By 48 h, the keratocytes on glass still retained the spread morphology but began developing small branches, while keratocytes on monomeric collagen began to elongate the branches formed earlier. From 72 h to 120 h, the keratocytes, regardless of topography, proceeded to further elongate the branches formed and displayed a stellate phenotype.

When keratocytes were cultured on glass (Figure 6D), monomeric collagen (Figure 6E), or aligned collagen fibrils (Figure 6F) coated with fibronectin, we observed differences in their morphology and spreading dynamics as compared to those without fibronectin. In general, the fibronectin coating caused keratocytes to exhibit a spread morphology for a longer period and delayed the formation of branches (see Supplementary Videos S5–S7). On the fibronectin-coated glass substrate, branching first started between 48 and 72 h, while on the fibronectin-coated monomeric collagen substrates and the aligned collagen fibrils, branching first started between 24 and 48 h.

**Figure 7:**
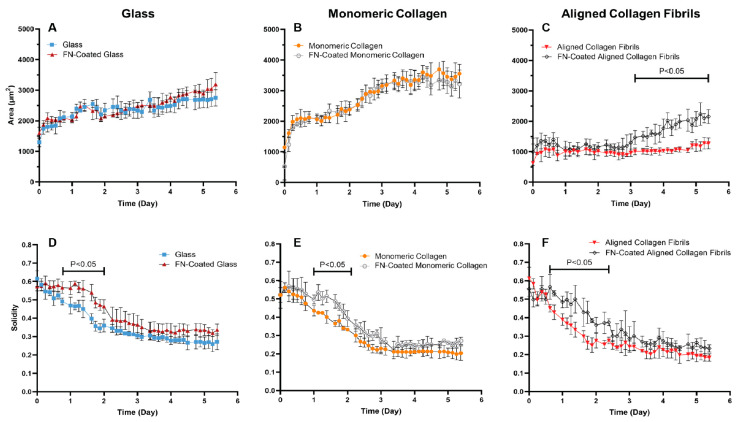
Effects of topography and ECM composition on keratocyte spreading and shape. Primary rabbit keratocytes (NRKs) were cultured in basal media on three different substrates (uncoated glass, monomeric collagen, or aligned collagen fibrils) without and with a fibronectin coating. Phase contrast time-lapse imaging was performed with a 10× objective with images collected every hour. Quantification of the changes in keratocyte spreading area and solidity on (**A**,**D**) glass, (**B**,**E**) monomeric collagen, and (**C**,**F**) aligned collagen fibrils. Each symbol represents the mean ± standard deviation for four experimental repeats and a total of 40 cells were analyzed. Data were analyzed using a two-way ANOVA followed by a Tukey post hoc test. A significance level of *p* < 0.05 was used for all the tests; ns *p* > 0.05.

In order to quantify the dynamic changes in morphology on the different substrates, we measured both the spreading area and the solidity. Solidity provided a measure of cell branching and overall shape complexity, with a lower solidity value representing cells with a larger number of branches. We chose to measure these two morphological parameters since previous studies have demonstrated that when keratocytes are activated by TGFβ they lose their dendritic morphology and adopt a more spread-out morphology (i.e., increased solidity) [48,49,50]. The use of solidity has previously been used to measure the reduction in branches and the more compact shape when a human corneal keratocytes cell line was activated by TGFβ [51]. Figure 7 demonstrates that there were significant differences in the spreading behavior of keratocytes on the different substrates. During the first ~3 h there was a significant increase in the spreading area on all three substrates (Figure 7A–C). After this initial increase, the spreading area of keratocytes on the aligned collagen fibril substrates appeared to remain relatively constant (~1000 μm^2^) over the next 5 days. In contrast, the spreading area of keratocytes on the uncoated glass increased during the first ~40 h and then plateaued at a surface area of ~2500 μm^2^, while the spreading area of keratocytes on the monomeric collagen-coated substrates increased steadily over the entire 5 days and plateaued at ~3500 μm^2^. The observation that cells had the smallest spreading area on the aligned collagen fibrils appears to be consistent with previous studies reporting that topographic features can reduce cell spreading [52,53]. Conversely the observation that cells had a higher cell spreading area on the collagen-coated glass as compared to the uncoated glass are consistent with reports that cell spreading is ECM-dependent [54,55].

When a fibronectin coating was deposited on either the uncoated glass or glass coated with monomeric collagen, there was no obvious difference in the cell spreading area over the entire 5 days (Figure 7A–C). In contrast, after about 3 days, the cells cultured on the fibronectin-coated aligned collagen fibrils increased their cell spreading area by almost 2-fold (e.g., 1000 to 2000 μm^2^). The reason why keratocytes increased their spreading area on the fibronectin-coated aligned collagen fibrils and not on fibronectin-coated glass or monomeric collagen is unknown and under further investigation.

As mentioned previously, we used solidity to quantify the degree of cell branching, where a decrease in solidity indicates the presence of more and longer branches extending from the cell body. Figure 7D–F shows that the solidity of keratocytes in the absence of fibronectin steadily decreased from ~0.6 to 0.2 over the 5-day culture period. Initially, the cells had a more rounded shape with few, if any, branches, resulting in the larger solidity values. Over time, the keratocytes formed multiple branches that stretched out in all directions, leading to a decrease in solidity. When keratocytes were cultured on fibronectin-coated surfaces, they formed fewer protrusions and thus the solidity remained relatively constant. On the fibronectin-coated glass, the solidity remained elevated and statistically different than the uncoated glass between the 18 and 48 h culture periods, while on fibronectin-coated monomeric collagen, the solidity was statistically different than the monomeric collagen alone for 24–48 h. Finally, when keratocytes were cultured on fibronectin-coated aligned collagen fibrils, the differences in solidity were different for 15–57 h.

**Figure 8:**
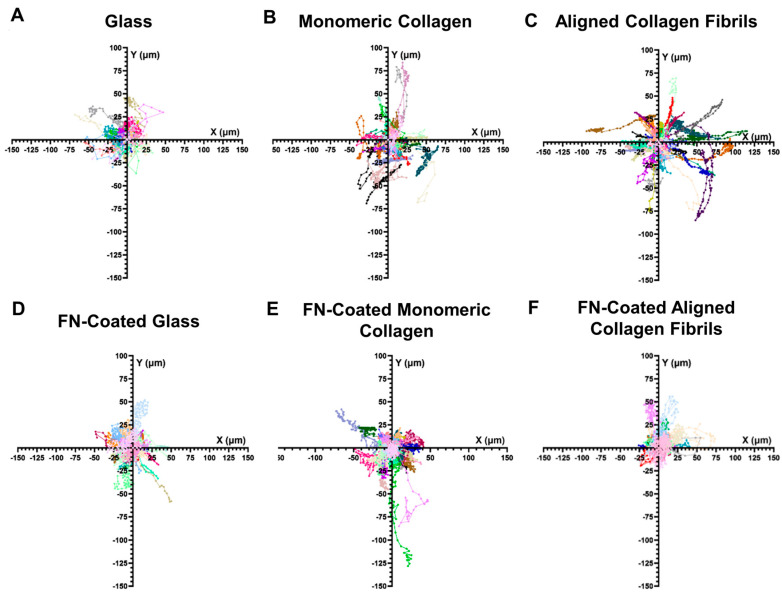
Effects of fibronectin coating and topography on keratocyte motility. Cell tracking plots displaying the individual cell paths from their origin as a function of distance in the x-y directions for 40 cells on glass, monomeric collagen, and aligned collagen fibrils without (**A**–**C**) and with (**D**–**F**) a fibronectin coating (*n* = 4 experimental replicates). Each colored line represents the actual spatial coordinates of one cell tracked over 120 h.

**Figure 9:**
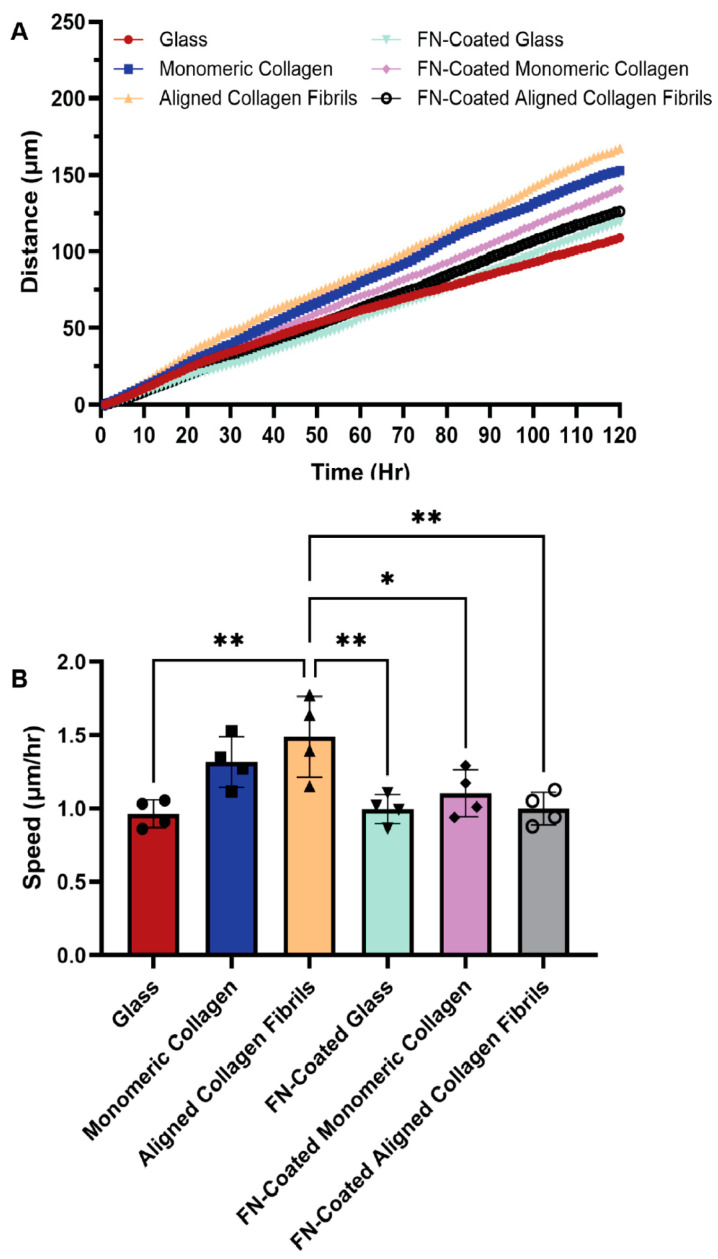
**Effect of topography and fibronectin coating on keratocyte accumulated distance and speed.** (**A**) Plot of mean accumulated distance traveled from 40 cells over 120 h on uncoated glass, monomeric collagen, and aligned collagen fibrils with and without a fibronectin coating. (**B**) Plot of average cell speed on the different substrates, from n = 4 experimental replicates. Data were analyzed using a two-way ANOVA followed by a Tukey post hoc test. A significance level of *p* < 0.05 was used for all the tests. * *p* < 0.05; ** *p* < 0.01.

In examining the time-lapse videos, it appeared that the motility of keratocytes on the fibronectin-coated substrates was limited. To determine whether there were subtle differences in keratocyte motility due to the fibronectin coating and/or differences in the substrate topography, we tracked their motility on the different substrates. Figure 8 shows representative cell tracking plots for 40 cells over 120 h on the six different substrates. When keratocytes were cultured on uncoated (Figure 8A) or fibronectin coated glass (Figure 8D), they migrated randomly and typically traveled only a small distance from their initial starting point with a maximum of ~50 μm. We observed a similar pattern of random and reduced motility on the monomeric collagen without (Figure 8B) and with a fibronectin coating (Figure 8E); however, a few cells did appear to be more motile as compared to the glass substrates. Keratocyte motility on the uncoated aligned collagen fibrils (Figure 8C) showed some motility in the direction of the fibrils (i.e., left to right) with some cells migrating a maximum of ~120 μm. However, the presence of a fibronectin coating on the aligned collagen fibrils appeared to reduce the directionality and distance traveled of the cells (Figure 8F). To further examine differences in cell motility, we made plots of the accumulated distance traveled (Figure 9A) and the cell speed (Figure 9B). Keratocytes on uncoated monomeric collagen and uncoated aligned collagen fibrils had an accumulated distance of 48% and 52% farther than on glass (Figure 9A) and had speeds of 1.25 and 1.5 μm/h, respectively. However, there was little difference in the distance traveled or speed of keratocytes on glass substrates with or without a fibronectin coating. In contrast, when fibronectin was present on both the monomeric collagen and aligned collagen fibril substrates, both the travel distance and speed of motility were reduced. This reduction in keratocyte motility on fibronectin-coated substrates was somewhat surprising since previous studies have reported that corneal fibroblast motility is increased on fibronectin [56,57] and that fibronectin serves as a temporary matrix over which corneal cells migrate during wound healing.

At this time, the exact reason why the fibronectin coating reduced cell protrusions and migration is unknown. The differences between our results and other studies are most likely due to differences in experimental protocols, such as the use of keratocytes vs. fibroblasts, human vs. rabbit cells, different fibronectin coating densities, and substrate surface chemistry. Previous studies have shown that the underlying surface chemistry of fibronectin-coated substrates can have a profound effect on the amount of fibronectin that adsorbs, the fibronectin conformation, the ability of integrins (e.g., α_5_β_1_) to bind to fibronectin, and the cellular response (e.g., proliferation and differentiation) [58,59,60]. It has also been reported that human fibroblast migration is faster on networks of fibrillar fibronectin than on globular fibronectin, and that increased cell remodeling of globular fibronectin compared to fibrillar fibronectin leads to slower migration [61]. In addition, it has been reported that the higher amounts of adsorbed fibronectin results in more prominent focal adhesions and reduces both cell polarization and membrane protrusions [62]. Thus, we suspect that the relatively high coating concentration of plasma fibronectin used in this study may be affecting the conformation of fibronectin and causing more prominent focal adhesions that are limiting both cell protrusions and migration. In support of this hypothesis are our previous observations that human corneal fibroblasts form more and larger focal adhesions on adsorbed fibronectin than on random or aligned collagen fibrils [14], and that cellular secretion of fibrillar fibronectin is higher on aligned collagen fibrils than on adsorbed fibronectin [55]. However, whether these observations are similar in rabbit corneal keratocytes are currently under investigation.

## 4. Conclusions

In this study we report a novel method for depositing aligned collagen fibrils onto glass coverslips in a 6-well format. Using this novel platform, we were able to investigate the dynamic behavior of corneal keratocytes in response to both biochemical cues (e.g., PDGF-BB, ECM composition) and biophysical cues (i.e., aligned collagen fibrils) over 5 days via continuous live-cell imaging. To demonstrate the versatility of this platform we measured dynamic changes in both cell morphology and cell motility. When primary rabbit keratocytes were cultured on aligned collagen fibrils in the presence of PDGF-BB, the keratocytes were three times more motile as compared to basal media and preferentially aligned parallel to the collagen fibril orientation. Conversely, when keratocytes were cultured on fibronectin-coated substrates we observed a reduced cell motility. Our analysis of cell morphology demonstrated that on fibronectin-coated substrates, keratocytes delayed the formation of cell protrusions which resulted in an elevated solidity over the initial 48 h. Although there was no difference in the spreading behavior when keratocytes were cultured on glass and monomeric substrates coated with fibronectin, we did observe that the spreading area of keratocytes on aligned collagen fibrils was significantly reduced as compared to monomeric collagen or uncoated glass. The observation that fibronectin transiently affected cell morphology but did not affect spreading area was surprising since these two properties are normally linked. However, studies with micropatterned substrates have shown that changes in cell morphology can occur while cell spreading is constant [63].

Previous studies have shown that the ECM topography not only induces morphological changes in corneal keratocytes [14,64,65], but also induces changes in their gene expression profiles [16,66,67]. However, gene expression profiles only provide a “snapshot” of the cell’s mRNA profile, and some gene expression changes are transient and can be easily missed if not collected at the right time. An advantage of continuous time-lapse imaging is that it allows investigators to detect the time frame where a cell undergoes a phenotype transition and to collect the cells precisely at that moment for gene expression analysis. We anticipate that combining our time-lapse imaging capabilities with paired gene expression data, innovative automated imaging tools, and advanced machine learning will allow us to eventually determine how morphological changes induced by combinations of topography and growth factors predict gene regulation and conversely, how gene expression regulates corneal keratocyte phenotype and function [68,69].

## Data Availability

Data is contained within the article and Supplementary Materials.

## Supplementary Materials

The following supporting information can be downloaded at https://www.mdpi.com/article/10.3390/mi17070783/s1. Figure S1: Effect of collagen solution pH and shear rate on collagen fibril alignment; Video S1: Dynamics of keratocyte morphology and alignment on aligned collagen fibrils in the presence PDGF-BB; Video S2: Keratocyte motility on aligned collagen fibrils in serum-free media; Video S3: Keratocyte motility on glass in serum-free media; Video S4: Keratocyte motility on monomeric collagen in serum-free media; Video S5: Keratocyte motility on fibronectin-coated glass in serum-free media; Video S6: Keratocyte motility on fibronectin-coated monomeric collagen in serum-free media; Video S7: Keratocyte motility on fibronectin-coated aligned collagen fibrils in serum-free media.
