# Supplementary material for: A Microwell Platform for Characterizing the Dynamic Response of Corneal Keratocytes to Biochemical and Biophysical Cues
Source: Micromachines (Basel). 2026 Jun 27;17(7):783. doi: 10.3390/mi17070783 (PMC13413648; doi:10.3390/mi17070783)
Supplement: Supplementary file 1 [file micromachines-17-00783-s001.zip › Tarik - Micromachines Paper - Supplementary Data.pdf]

Supplementary Data

for

A Microwell Platform for Characterizing the Dynamic Response of Corneal Keratocytes to Biochemical and Biophysical Cues

Tarik Z. Shihabeddin[1], Nathaniel S. Tjahjono[1], Divya Subramanian[1], Abbas Rizvi[1], Miguel Miron-Mendoza[2], Victor D. Varner [1,3], and David W. Schmidtke [1,3,*]

[1] Department of Bioengineering, University of Texas at Dallas, Richardson, TX 75080, USA; tarik.shihabeddin@utdallas.edu (T.Z.S.); nathaniel.tjahjono@utdallas.edu (N.S.T.); divya.subramanian@utdallas.edu (D.S.), abbasanengineer@gmail.com (A.R.); vdv@utdallas.edu (V.D.V.)

[2] Department of Ophthalmology, University of Texas Southwestern Medical Center, Dallas, TX 75390, USA; Miguel.Miron@UTSouthwestern.edu

[3] Department of Biomedical Engineering, University of Texas Southwestern Medical Center, Dallas, TX 75390, USA

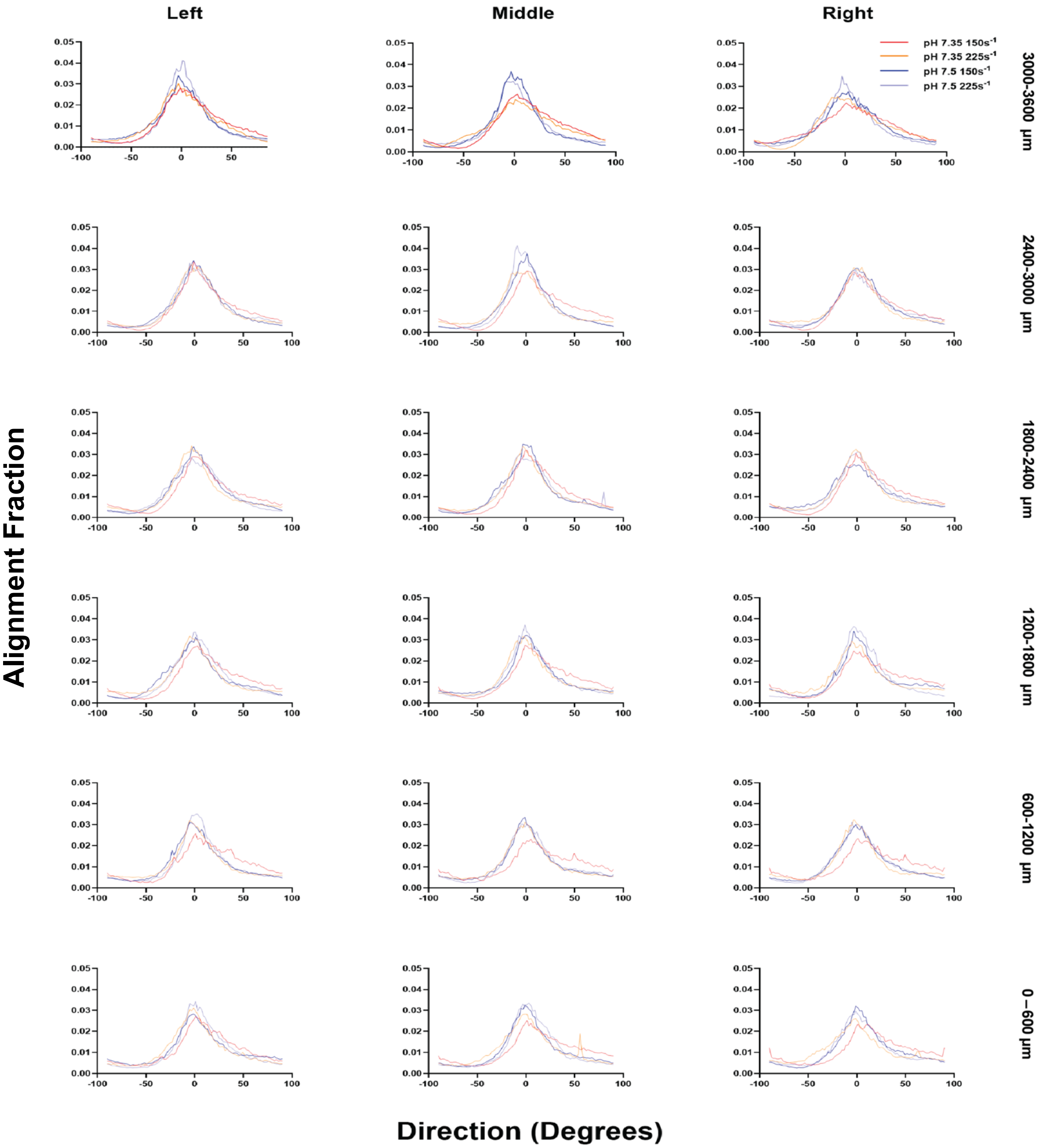

Figure S1: Effect of Collagen Solution pH and Shear rate on Collagen Fibril Alignment. Representative histograms of the aligned collagen fibril directionally throughout the length of the microfluidic channel when collagen solutions of different pH were perfused at different shear rates.

Video S1: **Dynamics of keratocyte morphology and alignment on aligned collagen fibrils in the presence PDGF-BB.** Representative time-lapse movie of changes in keratocyte morphology and alignment when cultured on aligned collagen fibrils in the presence of 50 ng/mL PDGF-BB for 120 hours.

Video S2: Keratocyte motility on aligned collagen fibrils in serum-free media. Representative time-lapse movie of changes in keratocyte morphology and motility when cultured on aligned collagen fibrils in serum-free media for 120 hours.

Video S3: Keratocyte motility on glass in serum-free media. Representative time-lapse movie of changes in keratocyte morphology and motility when cultured on Aquasil-coated hydrophobic glass in serum-free media for 120 hours.

Video S4: Keratocyte motility on monomeric collagen in serum-free media. Representative time-lapse movie of changes in keratocyte morphology and motility when cultured on monomeric collagen in serum-free media for 120 hours.

Video S5: Keratocyte motility on fibronectin-coated glass in serum-free media. Representative time-lapse movie of changes in keratocyte morphology and motility when cultured on fibronectin-coated glass in serum-free media for 120 hours.

Video S6: Keratocyte motility on fibronectin-coated monomeric collagen in serum-free media. Representative time-lapse movie of changes in keratocyte morphology and motility when cultured on fibronectin coated monomeric collagen in serum-free media for 120 hours.

Video S7: Keratocyte motility on fibronectin-coated aligned collagen fibrils in serum-free media. Representative time-lapse movie of changes in keratocyte morphology and motility when cultured on fibronectin coated aligned collagen fibrils in serum-free media for 120 hours.
